# Effects of Electrode Position on Spatiotemporal Auditory Nerve Fiber Responses: A 3D Computational Model Study

**DOI:** 10.1155/2015/934382

**Published:** 2015-02-10

**Authors:** Soojin Kang, Tanmoy Chwodhury, Il Joon Moon, Sung Hwa Hong, Hyejin Yang, Jong Ho Won, Jihwan Woo

**Affiliations:** ^1^School of Electrical Engineering, Biomedical Engineering, University of Ulsan, Ulsan 680-749, Republic of Korea; ^2^Department of Otorhinolaryngology-Head and Neck Surgery, Samsung Medical Center, Sungkyunkwan University, Seoul 330-714, Republic of Korea; ^3^Department of Audiology and Speech Pathology, University of Tennessee Health Science Center, Knoxville, TN 37996, USA

## Abstract

A cochlear implant (CI) is an auditory prosthesis that enables hearing by providing electrical stimuli through an electrode array. It has been previously established that the electrode position can influence CI performance. Thus, electrode position should be considered in order to achieve better CI results. This paper describes how the electrode position influences the auditory nerve fiber (ANF) response to either a single pulse or low- (250 pulses/s) and high-rate (5,000 pulses/s) pulse-trains using a computational model. The field potential in the cochlea was calculated using a three-dimensional finite-element model, and the ANF response was simulated using a biophysical ANF model. The effects were evaluated in terms of the dynamic range, stochasticity, and spike excitation pattern. The relative spread, threshold, jitter, and initiated node were analyzed for single-pulse response; and the dynamic range, threshold, initiated node, and interspike interval were analyzed for pulse-train stimuli responses. Electrode position was found to significantly affect the spatiotemporal pattern of the ANF response, and this effect was significantly dependent on the stimulus rate. We believe that these modeling results can provide guidance regarding perimodiolar and lateral insertion of CIs in clinical settings and help understand CI performance.

## 1. Introduction 

A cochlear implant (CI) is a medical prosthesis used to restore human auditory function. The electrodes of the CI are surgically placed into the scala tympani in the cochlea in order to deliver electric stimuli to excite the auditory nerve fibers (ANFs). Although the advances made over the past two decades have resulted in good CI speech recognition, this technology is still associated with a lack of music perception and speech recognition in noisy environments. Thus, many research groups have studied the signal processing [1, 2], surgical approach [3], and electrode design [4, 5] of CIs in order to improve CI performance. Another such effort is the ongoing research on the effects of electrode position. Perimodiolar electrode arrays have been reported to yield a lower threshold compared to electrodes located close to the outer wall [6–8]. When an electrode is located closer to the modiolus, less current is required to excite the ANFs. Mino et al. (2004) reported on the effects of electrode-to-fiber distance on spatiotemporal patterns of spike initiation using an ANF multicompartment model, and the results of this computer model may support and aid in the understanding of the physiological studies that have examined the effects of electrode position [9]. As the electrode-to-fiber distance increases, spike jitter, which is defined as the standard deviation of the spike latencies, is also increased; and great spike jitter could characterize the ensemble response of ANF fibers [10]. However, the effects of electrode position on the spatiotemporal response to pulse-train stimuli are still unclear. Furthermore, despite the modeling results of the variation of electrode-to-fiber distance, the previously reported models have a drawback due to nonrealistic conditions being used. For example, Mino et al. (2004) assumed the ANF as a simple straight shape in a homogeneous space. Therefore, we believe that a more realistic model that can accurately stimulate neural activity is needed, as this would allow the results to be compared to physiological and clinical data and used in current design to improve CI performance.

The goal of this study was to investigate the effects of electrode position on spatiotemporal responses to a single pulse and low- and high-rate pulse-trains. To estimate spatially distributed intracochlear electric fields, we have developed a three-dimensional finite-element (3D-FE) cochlear model. The ANF model was based on realistic anatomical features of a typical cat ANF. We included stochastic kinetics of sodium and potassium ion channels and an adaptation component into the ANF model to simulate the stochastic ion channel processes and rate adaptation that are typically observed in response to electric pulse-trains. Finally, we evaluated the effects of electrode positions on spatiotemporal ANF responses by placing the stimulus electrode at the four different sites in the scala tympani.

## 2. Materials and Methods 

### 2.1. The ANF Model

**Figure 1:**
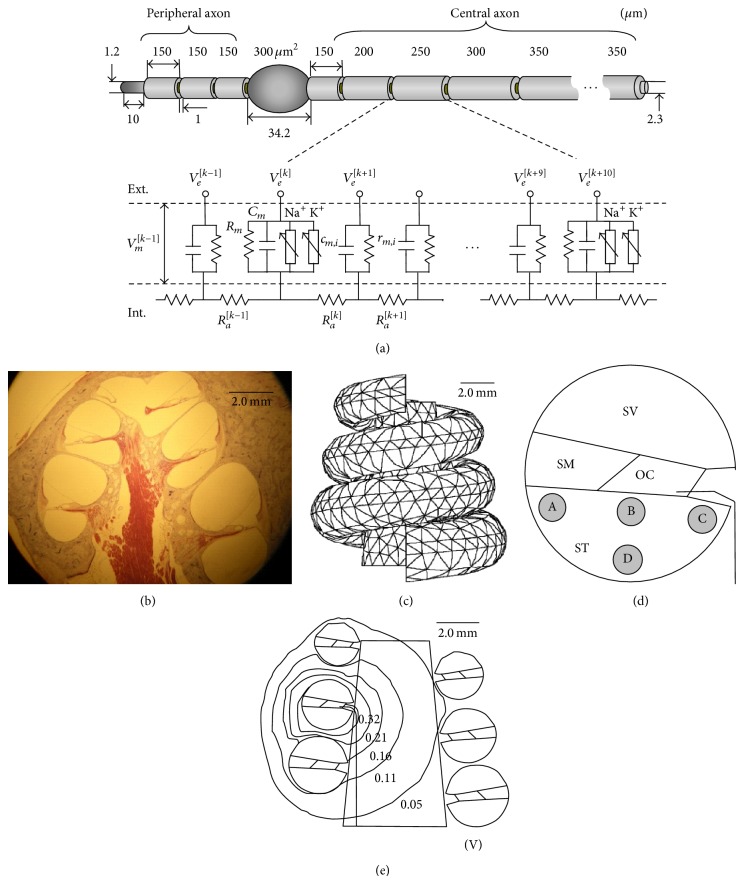
Summary of the 3D computational model. (a) Details of the anatomical features for the typical axonal model and the equivalent circuit. (b) Cross-sectional image of the cat cochlea used to create the finite-element mesh model. (c) The 3D finite-element mesh model with symmetrical rotation. (d) Simplified sectional drawing showing the positions of the stimulus electrodes (A–D) and the auditory nerve fiber. (e) Example of the electric field potential inside the cochlea. OC: organ of Corti, SM: scala media, ST: scala tympani, and SV: scala vestibuli.

The ANF model includes a cell body and peripheral and central axons, which are based on the known anatomical features of a cat [11, 12], such as 1.2 *μ*m diameter peripheral fibers, 2.3 *μ*m diameter central fibers, 1 *μ*m nodal gaps, and 1 *μ*m myelin thickness [13]. Each nodal section has an active node of Ranvier and nine passive nodes. Active (Ranvier) nodes include voltage-dependent Na and K channels that stochastically open and close based on a Markov jumping process using a channel-number tracking algorithm [9, 14]. The ion densities of the Na and K channels were 80/*μ*m^2^ and 45/*μ*m^2^, respectively, and their ion channel conductances (*γ*_Na_ and *γ*_K_) were 22.65 pS and 50 pS, respectively. All ion channel mechanisms were modified for the mammalian body temperature of 37°C.  Figure 1(a) shows the feature of a typical cat ANF, including a myelinated axon, node of Ranvier, cell body, and the equivalent circuit diagram. The transmembrane potential at *k*th compartment, *V*_*m*_^[*k*]^[*t*], is described in terms of nodal capacity, *C*_*m*_, nodal resistance, *R*_*a*_, and the applied electric potential, *V*_*e*_^[*k*]^[*t*] (1). A ball electrode was used to provide electrical stimulation, and the electric potentials at the *k*th nodal position were computed using a 3D-FE model as follows:

(1) −Vmk+1t−VmktRak+1,kt−Vmkt−Vmk−1tRak,k−1t+CmkVmkt+Δt−VmktΔt+VmktRmkt+Iionkt =Vek+1t−VektRak+1,kt−Vekt−Vek−1tRak,k−1tIion[k]t=γKNn4t,kVt,k−EK(t,k)+γNaNm3h1t,kVt,k−ENa.

The Na^+^ Nernst potential is 66 mV, the initial K^+^ Nernst potential is −88 mV, and the changes on the basis of efflux K^+^ current are as follows:

(2) $$\begin{array}{c} E_{\text{K}}\left(t,k\right)=\frac{RT}{F}\ln\left(\frac{\left[{\text{K}}_{\text{ext}}^{+}\right]\left(t,k\right)}{\left[{\text{K}}_{\text{in}}^{+}\right]}\right),\\ \Delta \left[{\text{K}}_{\text{ext}}^{+}\right]\left(t,k\right)=\overset{t}{\underset{i=0}{\sum }}I_{\text{K}}\left(i,k\right)\times g\left(t\Delta t-i\Delta t\right), \end{array}$$

where *R* is the universal gas constant, *T* is the temperature, *F* is the Faraday constant, and *g*(*t*) is the decay function. This modification produces accumulation of [K_ext_^+^] and changes the Nernst potential. The K current leakage mechanism alters the membrane voltage and simulates spike rate adaptation [15].

The partial differential equation of (1) was solved using the Crank-Nicholson method [9]. Details of the model parameters have been described in previous studies [12, 15].

### 2.2. 3D-FE Cochlear Model

A 3D-FE model was created on the basis of a midmodiolar section of the cat cochlea.  Figure 1(b) shows one midsection image obtained by embedding the cochlea in celloidin, sectioning at a 20-micron thickness and staining with hematoxylin and eosin. This section image was used to construct a simple 3D spiral model using finite-element software (ANSYS Inc., USA) (Figure 1(c)). The bounded regions were digitized to create finite-element meshes. The cochlea was segmented based on the tissue types of the scala vestibuli, scala tympani, scala media, organ of Corti, membrane tissue, modiolus, and nerve tissue. The peripheral nodes of the ANF were positioned within the organ of Corti, whereas the cell body was positioned within the bony compartment [8, 16, 17].  Table 1 lists the conductivity of each tissue [8]. The values were modified from the original values so that the model could produce a plausible relative spread of 3.0–6.0%, as observed in a previous cat experiment [18]. Each of the cross-sectional turns was connected by spiraling in a radial direction as seen in Figure 1(c).

We moreover modeled a simple ball electrode of 0.45 mm diameter as a stimulus electrode and placed it at the second turn of scala tympani in the cochlea.  Figure 1(d) shows a simplified cross section to identify the four different electrode sites of A to D as follows: “A,” outer wall; “B,” underneath the peripheral dendrite; “C,” close to the modiolus; “D,” middle of the scala tympani.

The 3D-FE program generated 3D meshes to solve the electric field of *V*_*e*_^[*k*]^[*t*] at each *k*th compartment of the ANF model and at specific time [*t*] (Figure 1(e)). Finally, the electric field data, *V*_*e*_^[*k*]^[*t*], is exported to (1) of the ANF model to simulate the ANF response to the electric stimulus of *V*_*e*_.

### 2.3. Simulation

The 3D-FE modeling and computation was performed on a workstation computer (Dell Precision 7500; Dell Inc., USA). After computing the electric field *V*_*e*_^[*k*]^[*t*], the data were employed to simulate the ANF response. The ANF modeling program was developed using Matlab (Mathworks, USA), and the numerical calculation was performed on a PC with a sample step, Δ*t* (in Equation (1)), of 1 *μ*s. The simulated stimulus was either a single rectangular biphasic pulse or pulse-trains presented at rates of 250 (low rate) and 5000 (high rate) pulses/s. The pulse-train stimuli consisted of rectangular biphasic pulses (cathode first) with a phasic duration of 40 *μ*s. The pulse-train duration was 200 ms and the amplitude was kept constant. The amplitude of a single pulse and pulse-trains for each electrode case was chosen to produce a plausible input/output response curve. A total of 100 repeated single-pulse stimulations and 30 repeated pulse-train stimulations were presented, and all model parameters were reset to the initial values before starting each simulation. The transmembrane potentials recorded at the 16th central node of Ranvier (C16) were used to calculate the response characteristics. The firing efficiency (FE) was computed as the ratio of the number of spikes to the number of sweeps. The threshold was defined as the current level that elicits an FE of 0.5. Responses to the electric pulse-train were characterized by a post-stimulus-time histogram (PSTH). The Na ion currents of all nodes of Ranvier were saved to explore the site of excitation. The spike-initiated node was defined by determining the minimum time in which the Na ion rushes into the intracellular space [19].

## 3. Results 

### 3.1. Responses to a Single Biphasic Pulse

**Figure 2:**
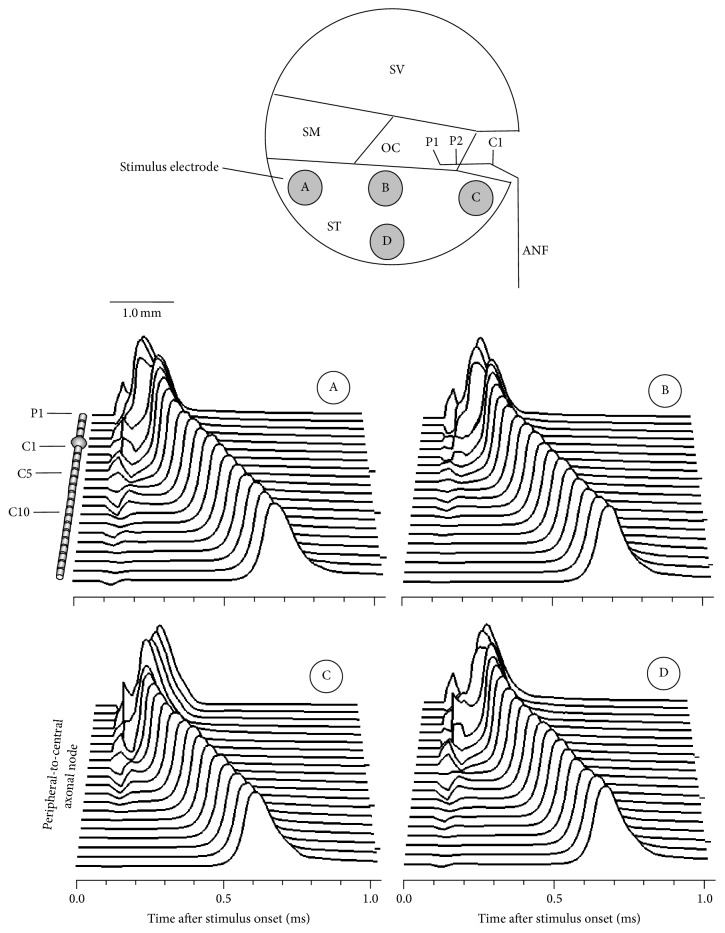
Example of transmembrane potentials in response to a single biphasic pulse from 4 different electrodes (A–D). The current level was set to evoke a firing efficiency of 0.5. ANF: auditory nerve fiber, C: central node, P: peripheral node, OC: organ of Corti, SM: scala media, ST: scala tympani, and SV: scala vestibuli.

Figure 2 shows examples of transmembrane potentials recorded from each node of Ranvier. In each case, the electrode was positioned at four different locations (A, B, C, and D). The membrane potential was plotted as a function of time after stimulus onset. The ordinate represents the peripheral-to-central axonal node. A single rectangular biphasic pulse (40 *μ*s/phase) was used and the stimulus level was chosen according to the threshold. In more centrally excited cases, the spike was found to be initiated at more central sites. The spike in case A was initiated at the peripheral node (P2), whereas the spike in case C was initiated at the central node (C2). All spikes propagated from the excitation node. The propagation velocity was 12.1 m/s, which is similar to the values observed in animal data [20]. The spikes in case C typically propagated to both the peripheral and central directions, supporting the previous observations regarding antidromic and orthodromic responses [21, 22].

**Figure 3:**
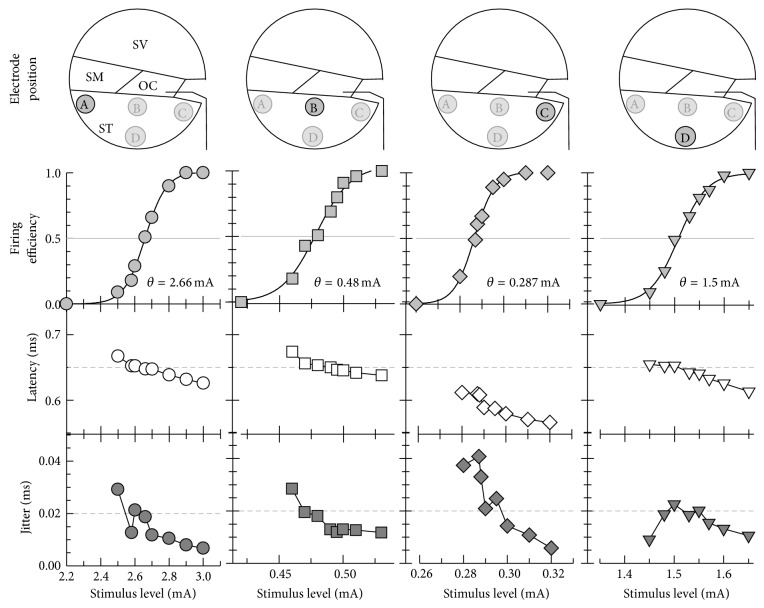
Comparison of responses to a single biphasic pulse for electrodes A (circle), B (rectangle), C (diamond), and D (triangle). The firing efficiency, mean latency, and jitter in response to 100 identical single pulses at each current level are plotted as functions of the stimulus level. The threshold (*θ*) is defined as the level evoking a firing efficiency of 0.5. OC: organ of Corti, SM: scala media, ST: scala tympani, and SV: scala vestibuli.

The electrode positions were found to have a significant effect on the site of excitation and influence the spike latency.  Figure 3 shows model responses to 100 repeated presentations. The FE (second row), mean latency (third row), and jitter (fourth row) were plotted as functions of the stimulus level. The mean latency and jitter were defined as the mean value and standard deviation of the spike latencies, respectively. The first row of each column shows the position of stimulus electrodes A–D. The FE was fitted to the sigmoid function to estimate the threshold (0.5 FE). The threshold for the electrode position close to the modiolus (C) was lower than those for the electrode positions near the peripheral node (A, B, and D). The threshold for the electrode A was relatively high. The mean latency and jitter decreased along with an increasing stimulus level, and these results are consistent with previous animal ANF data [23]. Moreover, the mean latency for electrode C was found to be shorter, owing to the fact that the spikes were centrally initiated.

The relative spread (RS) was used to measure the dynamic range [24] (Table 2). The RS values ranged from 0.028 to 0.041. Electrode C, which was close to the modiolus, produced the lowest RS, whereas electrode A near the outer wall produced the highest RS. As seen in Table 2, electrodes A and B showed benefits in terms of a higher RS, and electrode C produced lower thresholds in response to a single pulse.

### 3.2. Site of Excitation

**Figure 4:**
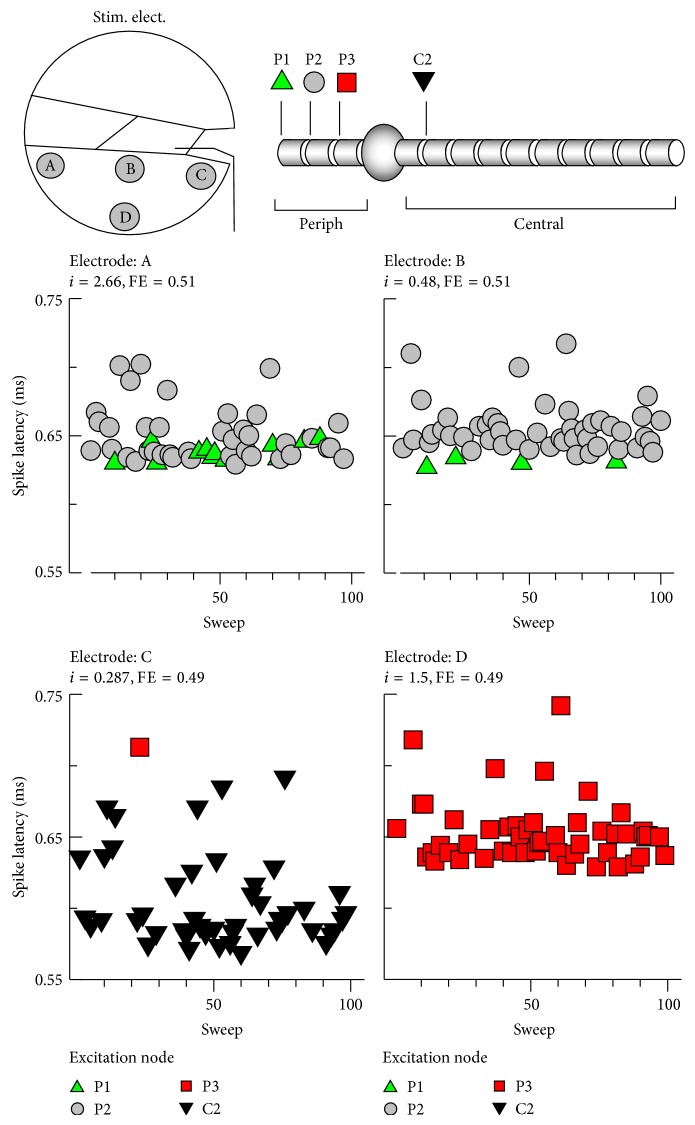
Spike latency across sweeps. Each symbol represents an excitation node. The stimulus level for each electrode (A–D) was chosen to elicit a 0.5-firing efficiency (FE) response. P: peripheral node; C: central node.

**Figure 5:**
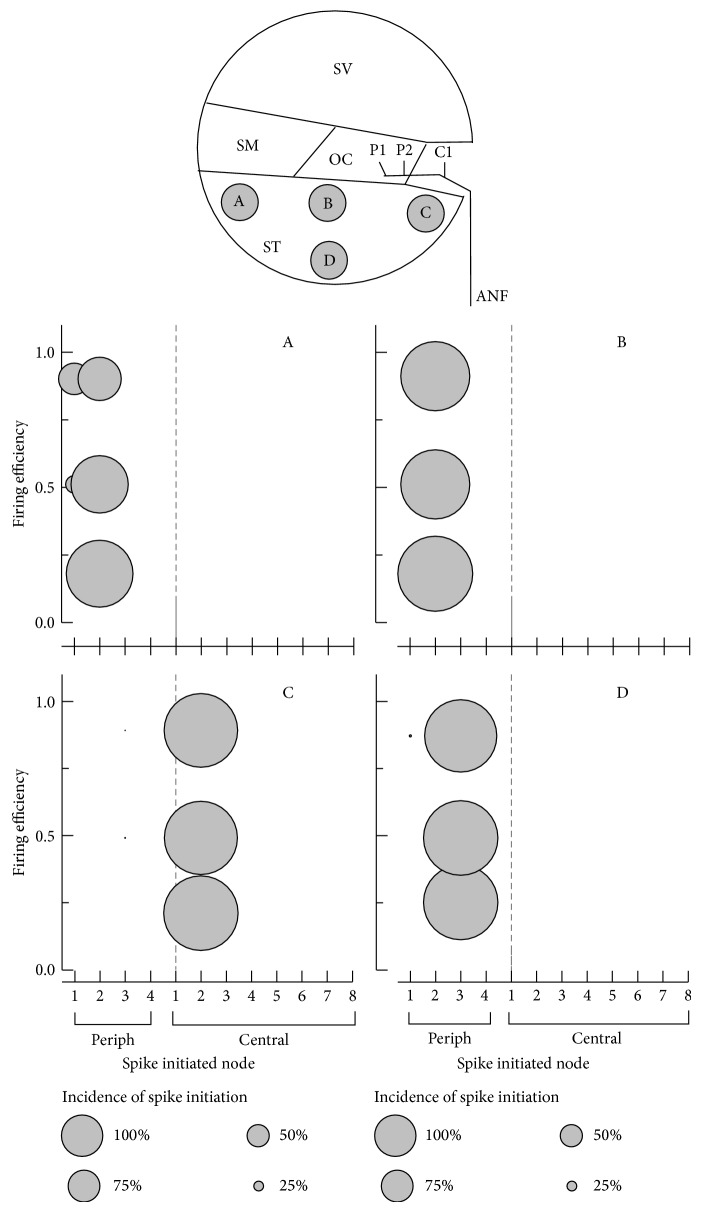
Incidence of spike initiation in response to a single pulse plotted for three different stimulus levels. The stimulus level was chosen to elicit firing efficiencies of 0.2, 0.5, and 0.9. The bubble size represents the incidence (%) of spike initiation. ANF: auditory nerve fiber, SM: scala media, ST: scala tympani, SV: scala vestibuli, periph/P: peripheral node, and central/C: central node.

The electrode position could influence the excitability of the ANF node. As seen in Figure 2, the sites of excitation could be either the peripheral node or central node, depending on the electrode position.  Figure 4 shows the details of the excitation node for the four different electrode positions. Each stimulus level was chosen to produce 0.5 FE. Each panel showed spike latencies across the sweeps for each electrode position. For electrode A, the spikes were initiated at either the P1 or P2 node; for electrode B, the spikes were initiated at either the P1 or P2 node; for electrode C, the spikes were initiated at either the P3 or C2 node; and for electrode D all spikes were initiated at the P3 node, indicating that the electrode position clearly influenced the site of excitation. ANFs stimulated using the electrode close to the modiolus were excited at the central node, whereas electric stimulation using the electrode at the lateral wall of the cochlea generated spikes that were initiated at the peripheral node. The mean latencies for A–D were 0.648, 0.653, 0.610, and 0.652 ms, respectively. These results suggest that more centrally initiated spikes resulted in shorter latency. The incidences of spike initiation for various stimulus levels are plotted in Figure 5. The stimulus levels were chosen to elicit FEs of 0.2, 0.5, and 0.9. For electrodes B–D, increasing the stimulus level of a single pulse did not influence the incidence of spike initiation.

### 3.3. Responses to Pulse-Trains

**Figure 6:**
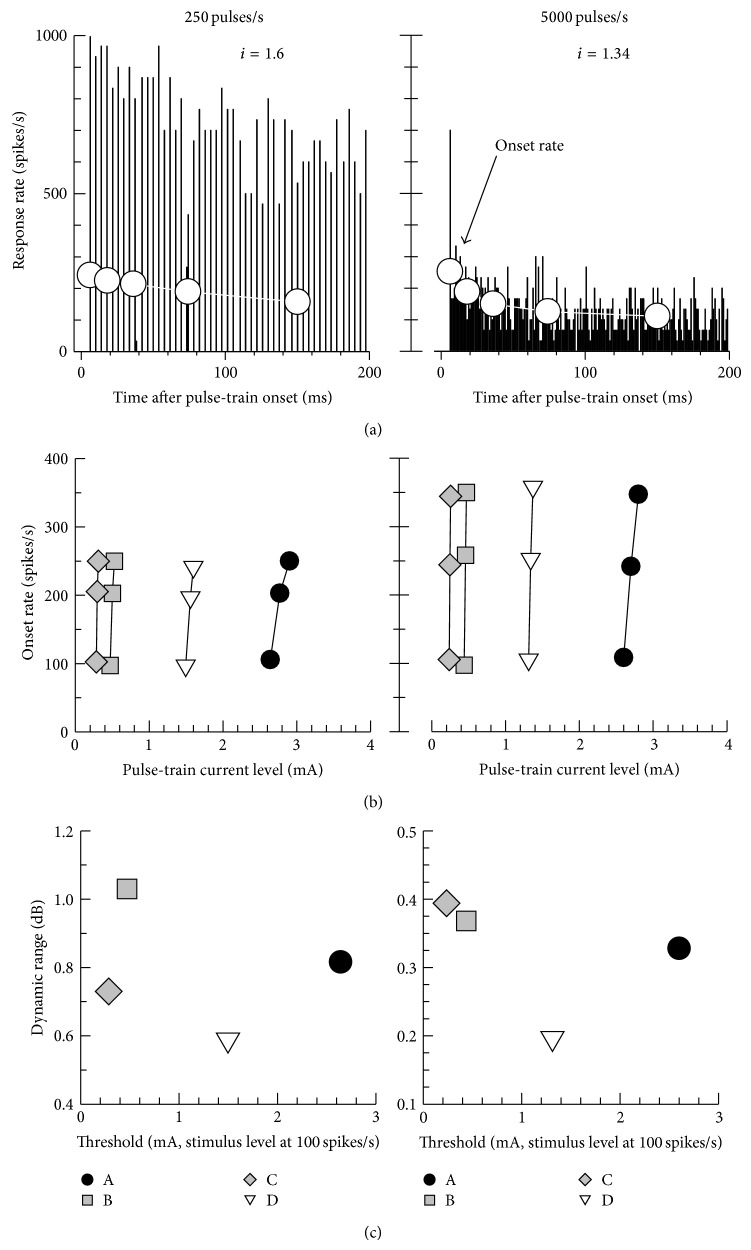
Responses to low-rate (250 pulses/s) (left column) and high-rate (5,000 pulses/s) (right column) pulse-trains. (a) Examples of post-stimulus-time histograms with the 1 ms bin widths vertical bar and wider bin (0–12, 12–24, 24–48, 48–100, and 100–200 ms) circles. (b) Onset (0–12 ms) response rates for electrodes A–D are plotted as a function of the pulse-train level. (c) The dynamic ranges versus the thresholds for the low- and high-rate pulse-trains are plotted. The threshold was defined as the stimulus level that elicited a 100-spike/s onset response rate. The dynamic range was calculated from curve (b) of the onset rate versus current level.

Examples of PSTHs in response to 250- and 5000-pulse/s pulse-trains are plotted in Figure 6(a). The low- and high-rate electric pulse-trains were presented at the levels of 1.6 and 1.34 mA, respectively, using electrode D. In each PSTH panel, both the 1 ms smaller bin (line-bar) and the wide bins (0–12, 12–24, 24–48, 48–100, and 100–200 ms) are plotted to show the response alternation and rate adaptation. The response rates for the wide bins are plotted at the midpoint of each bin range (6, 18, 36, 74, and 150 ms, resp.). The response rate was found to clearly decrease across time in both cases, indicating a rate adaptation similar to that observed in cat ANFs [25]. The onset rate-level functions for the 250- and 5000-pulse/s stimuli are plotted in Figure 6(b). For each case, stimulus levels to elicit approximately 100-, 200-, and 250-spike/s onset rates for 250-pulse/s and 100-, 250-, and 350-spike/s onset rates for 5000-pulse/s stimuli were chosen. The threshold for the pulse-train stimuli case was defined as the stimulus level that evoked a 100-spike/s onset (0–12 ms) rate [25] and was estimated by fitting a line to the rate-level data. The dynamic range for pulse-train stimuli was defined as the dB range that evoked an onset rate of 100–250 spikes/s.  Figure 6(c) plots the dynamic range versus threshold for the different electrode positions. Dynamic range versus threshold data for low-rate (250 spikes/s) pulse-trains showed a similar tendency to the RS versus threshold data for a single pulse. For example, the dynamic range for electrode A was higher than that of other electrodes. However, the high-rate (5000 spikes/s) pulse-train showed distinctly different results. For high-rate pulse-train cases, electrode C produced a wider dynamic range and lower threshold compared to electrodes A, B, and D.

**Figure 7:**
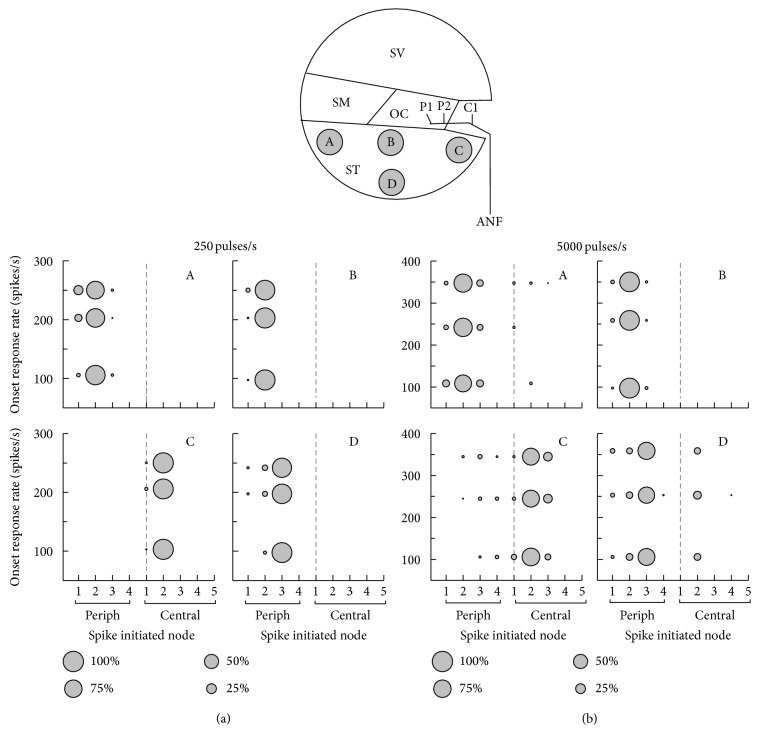
The incidence of spike initiation in response to 250- (first two columns) and 5,000- (last two columns) pulse/s pulse-trains. The pulse-train duration was 200 ms. The pulse-train levels were chosen to elicit onset (0–12 ms) response rates of 100, 200, and 250 spikes/s for low-rate stimuli and 100, 250, and 350 spikes/s for high-rate stimuli. An abscissa represents a spike-initiated node. The incidence (%) of spike initiation is represented by bubble size. ANF: auditory nerve fiber, SM: scala media, ST: scala tympani, SV: scala vestibuli, periph/P: peripheral node, and central/C: central node.

Figure 7 shows the incidence of spike initiation for the 250- and 5,000-pulse/s pulse-trains. The stimulus level was denoted as the onset (0–12 ms) response rate. To allow comparisons across the four electrode cases, the stimulus levels were chosen to elicit 100, 200, and 250 spikes/s for 250-pulse/s stimuli and 100, 250, and 350 spikes/s for 5,000-pulse/s stimuli. For the 250-pulse/s stimuli case, the sites of excitation were similar to those in response to a single pulse (Figure 5). However, for the 5,000-pulse/s high-rate stimulation, the distribution of the initiated nodes was clearly different from that in response to a single pulse; that is, it was more widely distributed. Typically, for electrode C, the spikes were initiated between P2 and C3; as the stimulus level increased, the spike was initiated at the more central node, C3. These results indicate that the electrode positions, as well as the stimulus rate, clearly influenced the spatio-ANF responses.

**Figure 8:**
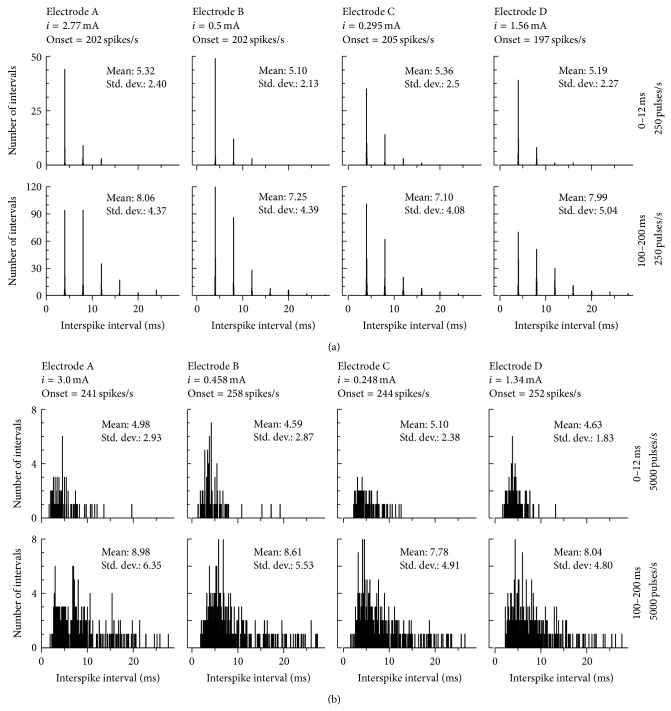
Interval histograms for different electrodes (A–D, column) in response to 250- (first and second rows) and 5,000-pulse/s (third and fourth rows) pulse-trains. At each pulse rate, the interval histogram was analyzed over either an onset (0–12 ms) or steady-state (100–200 ms) period. The stimulus levels were chosen to elicit response rates of 200 and 250 spikes/s for 250- and 5,000-pulse/s stimuli, respectively. The bin width was set to 50 *μ*s.

Figure 8 shows interval histograms for rate stimuli of 250 and 5000 pulses/s. The stimulus level for each electrode case was chosen to produce an onset response rate of 200 spikes/s for 250-pulse/s pulse-trains and 250 spikes/s for 5,000-pulse/s pulse-trains. For low-rate stimulation, phase-locks of the spike to pulse-train stimulation were observed, and the overall trends for all 4 electrode cases were similar. However, the intervals for high-rate stimulation were broadly distributed. These trends of low- and high-rate stimulation were similar to those observed in cat ANFs [26].

## 4. Discussion 

### 4.1. Summary and Comparison with Physiological and Clinical Studies

This study investigated the effects of electrode position on ANF responses to either a single pulse or low/high-rate pulse-trains. To evaluate the spatiotemporal responses, the jitter, latency, inter-spike interval, and spike initiation for each electrode case were compared. As seen in Table 2, the stimulation type, either single-pulse or pulse-train stimuli, influenced the effects of the electrode position on the neural response. At a single-pulse presentation, electrode B, which was close to the peripheral axon, produced a longer mean latency and higher threshold, while electrode C, which was close to the central axon, produced a shorter mean latency and lower threshold. These trends are consistent with single-fiber measures in cat ANFs [23, 27]. Thus, the simulated results clearly showed that different sites of excitation resulted in different responses, as hypothesized based on previous physiological studies.

The stochasticity of the ANF responses to the pulse-train was different to that to a single pulse. The distribution of initiation nodes in response to the pulse-train is wider than that in response to a single pulse. The overall response trends to the low- and high-rate pulse-trains were similar to previously reported physiological data [26, 28]. Miller et al. (2008) reported significant across-fiber variability in the interval histogram data from their animal study and discussed the possibility of an effect of various distances between the stimulus electrode and ANF [26]. The current results suggest that their hypothesis is likely correct and that these findings likely resulted mainly from the different dispersions of the site of excitation depending on the electrode position. Electrode C, which modeled a perimodiolar electrode in the CI, produced a greater spike jitter and could hence produce more ensemble responses. A previous clinical study reported that the pitch discrimination ability for the perimodiolar contour array was improved compared to that for the straight array [4] and that the electrode-to-modiolus distance significantly increased the threshold in CI users [29]. The result of the present study could account for and is consistent with these previous clinical data.

### 4.2. Future Works

In this study, typical-diameter (2.3 *μ*m) ANF and a simple 3D model of the cochlea were used. It has been reported that the diameters of ANFs range from 1.2 *μ*m to 4.6 *μ*m [11] and that the fiber diameter significantly influences the response to electrical stimuli. Thus, simulation using various anatomical features of ANFs may strengthen the current results. Furthermore, the present model could potentially also be used to simulate effects of specific biological or anatomical conditions such as demyelination on spatial-temporal responses to electric stimuli. Previous modeling studies have shown that the electric field can be controlled not only by the electrode position but also by the stimulus strategy and electrode configuration [7, 30–32]. We expect that the current work can be used to extend the previous modeling results and further explore the effects of the stimulus strategy on the spatial and temporal responses to high-rate and low-rate pulse-trains.

## Figures and Tables

**Table 1:** Conductivities of the components in the 3D cochlear model^*^.

Tissue	Conductivity (Ωm)^−1^
Scala tympani	1.43
Scala vestibuli	1.43
Scala media	1.67
Organ of Corti	0.012
Modiolus	0.156
Nerve tissue	0.3
Electrode	0.58*e*7

^*^Derived from Frijns et al. (1995) [7].

**Table 2:** Summary of the effects of electrode position on electrically evoked responses.

Stimulus		Electrode position				Figure
		A	B	C	D	
Single pulse	RS	0.041	0.040	0.028	0.031	Figure 3
	Threshold (mA)^*^	2.66	0.48	0.29	1.51	Figure 3
	Jitter (ms)	0.019	0.017	0.024	0.022	Figure 3
	Mean latency (ms)	0.65	0.65	0.58	0.65	Figure 3
	Initiated nodes	P1, 2	P1, 2	P3, C2	P3	Figure 5

Pulse-train (250 pulse/s)	Dynamic range (dB)	0.81	1.03	0.73	0.59	Figure 6
	Threshold (mA)	2.64	0.47	0.28	1.49	Figure 6
	Initiated nodes	P1, 2, 3	P1, 2	C1, 2	P1, 2, 3	Figure 7
	ISI mean (0–12 ms) (100–200 ms) ISI std. dev. (0–12 ms) (100–200 ms)	5.32 8.06 2.40 4.37	5.10 7.25 2.13 4.39	5.36 7.10 2.50 4.08	5.19 7.99 2.27 5.04	Figure 8

Pulse-train (5000 pulse/s)	Dynamic range (dB)	0.32	0.36	0.39	0.19	Figure 6
	Threshold (mA)^**^	2.60	0.43	0.23	1.31	Figure 6
	Initiated nodes	P1, 2, 3 C1, 2, 3	P1, 2, 3	P2, 3, 4, C1, 2, 3	P1, 2, 3, 4 C2, 4	Figure 7
	ISI mean (0–12 ms)^***^ (100–200 ms) ISI std. dev. (0–12 ms) (100–200 ms)	4.98 8.98 2.93 6.35	4.59 8.61 2.87 5.53	5.10 7.78 2.38 4.91	4.63 8.04 1.83 4.80	Figure 8

^*^Current level evoked 0.5-firing efficiency; ^**^current level evoked a 100-spike/s onset response; ^***^interspike interval at onset response of 250 spikes/s.

C: central, ISI: interspike interval, P: peripheral, RS: relative spread, and std. dev.: standard deviation.
